# Comments on ‘Lattice Boltzmann simulation of alumina-water nanofluid in a square cavity’ by Yurong He, Cong Qi, Yanwei Hu, Bin Qin, Fengchen Li and Yulong Ding

**DOI:** 10.1186/1556-276X-7-648

**Published:** 2012-11-24

**Authors:** Nor Azwadi Che Sidik, Arman Safdari

**Affiliations:** 1 Faculty of Mechanical Engineering, Universiti Teknologi Malaysia, UTM Skudai, Johor, 81310, Malaysia

**Keywords:** Lattice Boltzmann, Nanofluid, Volume fraction, Nusselt number

## Abstract

This work presents some comments concerning the paper entitled ‘Lattice Boltzmann simulation of alumina-water nanofluid in a square cavity’ by Yurong He, Cong Qi, Yanwei Hu, Bin Qin, Fengchen Li and Yulong Ding which was published in *Nanoscale Research Letters* in 2011. The comments are related to the numerical parameters and the computed results of average Nusselt number.

## Background

The authors of ‘Lattice Boltzmann simulation of alumina-water nanofluid in a square cavity’ [1] have used the conventional lattice Boltzmann scheme to analyse the heat transfer and flow characteristics of Al_2_O_3_-water nanofluid in a square cavity. They described the numerical methodology which implemented the double-population lattice Boltzmann scheme to predict both flow and thermal fields. In this paper, few comments on the numerical parameters and the computed results will be highlighted, followed by a conclusion and list of references.

### Numerical parameter

The authors have carried out a grid independence test using three grid sizes of 192^2^, 256^2^ and 300^2^ to predict the average Nusselt number at Ra = 8 × 10^5^ and 0% of volume fraction (page 5 of 8). The authors have chosen the 256^2^ grid size and claimed that this grid size gave a grid-independent solution. Since 0% of the volume fraction has been chosen, the condition can be referred as natural convection of pure water in an enclosure [2-5]. However, the rest of the predictions involved a more critical condition, such as the Rayleigh number being greater than 10^6^ with a 5% of volume fraction of Al_2_O_3_. Thus, it is extremely difficult to rely on the grid size that resulted from the grid independence test at such mild condition. In addition, the obtained average Nusselt number at a grid size of 256^2^ deviates about 4.5% from the benchmark results. Therefore, it can be concluded that this grid size is not suitable and can lead to wrong results as will be discussed in the ‘Numerical results’ section.

### Numerical results

The authors have stated that as the volume fraction is increased, the fluid becomes more viscous and the velocity of the flow in the enclosure decreases (page 7 of 8). At this point, the authors ignored the fact that the presence of nanoparticles stimulates the flow that resulted from high-energy transport through the flow associated with the irregular motion of nanoparticles [6-8]. This behaviour leads the thermal boundary layer to become thinner and the Nusselt number to increase [9]. Even though the authors have claimed that the Nusselt number decreases as the volume fraction increases, which was demonstrated in figure six of the said article, their results however are still questionable since they have been computed using inappropriate grid sizes.

## Discussion

The present commentary aimed at providing an in-depth discussion on the numerical methodology described in the work of He et al. [1]. The contribution of the present work is in correcting a number of mistakes made in an attempt to predict the heat and flow characteristics of nanofluids in enclosure.

## Conclusion

We recommend that the authors perform code validation by comparing with the well-known benchmark solution. We also suggest that the authors conduct a grid independence test on the most critical condition of their research case in order to comprehend the effect of grid size on the numerical solution.

## Competing interests

The authors declare that they have no competing interests.

## Authors’ contributions

AS conceived the study and checked the grammar of the manuscript. NACS drafted the manuscript. Both authors read and approved the final manuscript.
